# Mapping the Global Evolution and Research Directions of Information Seeking, Sharing and Communication in Disasters: A Bibliometric Study

**DOI:** 10.3390/ijerph192214878

**Published:** 2022-11-11

**Authors:** Hao Tan, Yuyue Hao

**Affiliations:** Key Laboratory of Advance Design and Simulation Technology for Special Equipments Ministry of Education, Hunan University, Changsha 410082, China

**Keywords:** communication behavior, information seeking, information sharing, bibliometrics, disaster management

## Abstract

This paper aims to grasp developments and trends in research on information communication, information seeking and information sharing in disasters during 2000–2021. By using bibliometrics software CiteSpace and VOSviewer, the development trends of publications, disciplinary, journals, institutions and regional cooperation are mapped. Keyword co-occurrence analysis is used to further identify the evolution of the research hot points and visualize the research orientation and frontier. The results indicate that the field of information communication in disasters has received growing attention from various disciplines. Results of institutions and regional cooperation show that worldwide cooperation is still lacking and needs to be strengthened in future studies in this field. The key findings are five main research orientations in this field based on keyword co-occurrence, which are public information coordination research, public information behavior and perception research, health information communication research, risk communication and social media research and information technology in emergency management. The findings of this paper can be helpful for academics and emergency managers in disaster information management and risk communication by giving them a comprehensive understanding of people’s information communication, seeking and sharing.

## 1. Introduction

Information communication in disasters shows great importance in improving rescue efficiency, owing to its impact on people’s decision-making and behavior in disasters. People are now living in an environment dominated by digital and social media [1]. How they seek, share and spread information in disasters has become a research topic of great concern and has been widely studied in many disaster domains, such as hurricanes [2,3], typhoons [4] and floods [5]. The value of effective information communication for saving lives and livelihoods in disasters has been recognized as the core factor for the success of disaster management [5]. Many cases of failure of emergency response due to poor communication illustrate the importance of disaster information dissemination research.

With the increase in worldwide attention to information seeking, information sharing and communication in disasters, the field has accumulated plenty of academic achievements. By overviewing the literature on disaster engineering, environmental science and information management, 50 studies that review the information behavior in disasters have been conducted, most of which focused on the information behaviors and communication pathway in emergency response [6,7,8,9], community preparedness in disasters [10,11] and risk communication [12,13,14,15]. In addition, information communication structures and related information and communications technology in emergency management are also discussed [16,17]. However, most of the existing studies study information communication behaviors from a single perspective of emergency management or disaster response. There is still a lack of systematic literature and theoretical framework for research on the information communication behaviors in disasters. This research topic involves many research fields, such as public opinion communication and disaster management, which makes it hard to sort out the current complex research status. It is necessary to integrate these studies over the past few decades into a systematic and comprehensive framework. It will be exceedingly helpful for scholars to understand the development of this discipline, to find the current research trends and hotspots and to conduct more valuable research of information communication in disasters in the future. Thus, there is an urgent need for an evaluation of research based on interdisciplinary literature data, integrating multidisciplinary perspectives and adopting bibliometric methods to record published research. This study aims to fill this gap via the bibliometric method. A quantitative performance description of current research in the field of information communication in disasters is presented first, including the social network among researchers, institutions and journals in this field. Then, co-occurrence network analysis is used to study the development status and research hotspots in this field by analyzing the co-occurrence network of keywords of publications. Finally, based on quantitative and qualitative analysis results, research focus and the trend of development of information communication behavior in disasters are identified.

This study presents a comprehensive overview of publications related to people’s information seeking, information sharing and information communication in the past two decades (2000–2021). By analyzing a bibliometric dataset of 1146 articles retrieved from the Web of Science (WoS), this paper presents a panorama of people’s information behavior in disasters and studies the research topics and trends in this field via bibliometric methods. The results of this study will help relevant researchers grasp the subject status and research trend of information communication behavior in disaster research and provide suggestions for their research direction in this field.

## 2. Materials and Methods

Bibliometrics is a statistical method that provides innovative insights into research trends in a specific field by analyzing the citations and contents of publications [18,19]. With the advantages of large-scale applicability, lower costs as well as perceived objectivity, the bibliometric method and bibliometric indicators have been widely recognized and applied by governments and institutions [18]. Compared with traditional peer review and expert judgment, the bibliometric method can provide quantitative indicators through the statistical analysis of academic achievements to ensure the objectivity of research conclusions. The bibliometric method helps researchers to acquire quantitative information from large amounts of data according to various bibliometric descriptors and indicators [20]. In addition, the use of bibliometric methods to provide research status and trends in a specific research field will help young researchers find future research directions [21]. Since the bibliometric method has been widely used in many disciplines related to emergency, such as emergency management [20], safety culture [22], information science [23] and behavioral science [24], this study uses two bibliometric methods, the co-occurrence network of keywords and the keyword clustering, to explore the knowledge structure and trend of people’s information behavior in disasters.

### 2.1. Data Retrieval

This paper conducts a quantitative bibliometric analysis and visualization of publications relating to people’s behavior of information seeking, information sharing and information communication in disasters through bibliometric methods based on the Web of Science (WOS) academic database. Web of Science (WoS) is one of the most authoritative scholarship databases which covers a wide variety of subjects in various disciplines. Meanwhile, WoS is also the standard tool for conducting extensive citation searching and bibliometric analysis, as it offers general and comprehensive citations [25].

In the literature-retrieval stage, the topic “information behavior” was defined as people’s actions to seek, share and spread information related to disasters. Next, we defined the range of disasters by referring to Valkengoed’s study on natural disasters [26] and Sugiura’s study on emergency disasters [27], including earthquake, hurricane, flood, tsunami, typhoon, wildfire, landslide, storm, fire and explosion and other major natural and man-made disasters of great concern. Therefore, the topics “(“disaster” OR “hazard” OR “emergency” OR “earthquake” OR “hurricane” OR “flood” OR “tsunami” OR “typhoon” OR “tornado” OR “wildfire” OR “landslide” OR “land slide” OR “storm” OR “fire” OR “explosion”) AND (“information seeking” OR “information sharing” OR “information communication”)” were used for retrieval. In addition, the publications of the medical surgery category are removed from the search results to focus on the target research field. This query produces 1146 records, including 674 papers from academic journals, 439 conference papers and other editorial materials and views. All search items are exported from WOS in “TXT” format with complete records and references to ensure that relevant research literature is fully covered as much as possible and duplicate literature is merged.

### 2.2. Data Analysis Method

The bibliometric analysis mainly includes two parts of data analysis [28,29]: (1) performance analysis to provide descriptive statistics on the distribution of journals, disciplines, cooperative networks of researchers and research institutions related to specific research fields and (2) co-occurrence network analysis to describe the network of research topics between publications according to the co-occurrence description of keywords or citations. Performance analysis aims to use the co-citation network among publication, authors and their institutions to grasp the general developmental and collaborative situation in a specific research field [24]. Keyword co-occurrence analysis assumes that words often occurring together are thematically similar. This method helps to describe the internal relationship and structure in a specific field and guides the researcher in finding the core content of the literature [30,31]. These two parts work together to constitute an objective overview of the current situation and development trend of a knowledge field.

Based on the first part, this study conducts the performance analysis of research on people’s information communication behavior in disasters to sort out the current situation of research and cooperation in this field and determine the influence of researchers and institutions in this field through quantitative indicators. In the second part, the keyword co-occurrence analysis was used to explore the emergence and evolution of research topics in the field. A further keyword clustering analysis was conducted to identify the main research orientations in the field of information communication in disasters.

### 2.3. Data Analysis Tools

In this study, the bibliometrics software CiteSpace (Version 6.1.R3) and VOSviewer (Version 1.6.18) are used for the analysis and visualization of publication data. VOSviewer, developed by van Eck and Waltman [32] at Leiden University, is a tool specifically designed for constructing and visualizing bibliometric maps and specializes in graphical representation [20]. It is used to extract the author information (name, country, institution) and keywords of publications and plot their correlation according to these key feature indicators [19,22]. In recent years, VOSviewer has been widely recognized and applied in the field of bibliometrics and knowledge mapping because of its simple and friendly interface design and reliable results. CiteSpace, developed by Chen [33] at Drexel University using Java language, is an information-visualization software based on citation-analysis theory. It can be used to explore citation hotspots and growth trends in specific research fields and plot the keyword timeline view [33]. By aggregating literature networks into several clusters and marking them with core keywords, it can help to explain the topics concerned by relevant researchers and the relationship between them [34].

## 3. Results

### 3.1. Performance Analysis

In this section, a comprehensive overview of studies of people’s information communication behavior in disasters was conducted by analyzing the performance of publications, disciplinary, journals, research institutions and regional cooperation of these 1146 articles.

#### 3.1.1. Publication Performance

**Figure 1:**
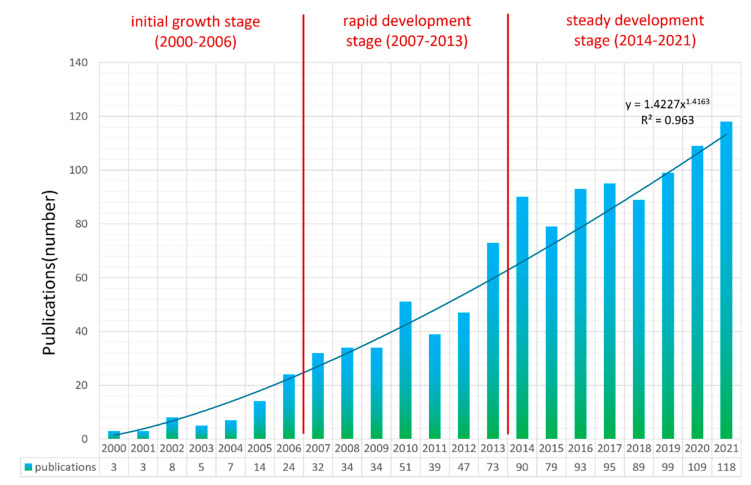
The total number of publications during 2000–2021. The number of annual publications and the three main stages in research on information communication in disasters are shown.

The trend of publications related to information communication in disasters in the past two decades is shown in Figure 1. The figure shows the number of papers published in the research field per year and carries out a power curve fitting regression analysis for the number of documents (*p* < 0.001, R^2^ = 0.9514). The attention of researchers on information seeking and sharing in disasters is increasing year by year. Based on the analysis of the annual distribution [35], the whole development process can be divided into three main stages: the initial growth stage (2000–2006), the rapid development stage (2007–2013) and the steady development stage (2014–2021). As shown in Figure 1, publications in the initial growth stage are very limited and it was not until 2006 that the annual publications exceeded 20. From 2007 to 2013, with the popularity of mobile Internet and multimedia channels, people’s behavior for research on information seeking, sharing and communication of disaster information has attracted more and more attention and the amount of literature in this field is also growing rapidly. Since 2014, publications in this research area have increased steadily and the annual number of publications issued reached over 100 in 2020.

**Figure 2:**
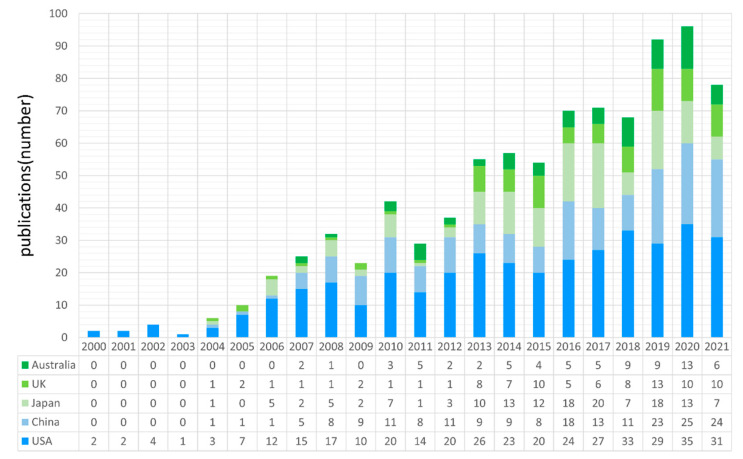
Publications in TOP 5 publication countries during 2000–2021. The number of annual publications of the five countries with the highest number of publications is shown.

From the perspective of countries, the growth trend of the five countries with the highest number of publications is highlighted in Figure 2. The table shows the annual publications of the five countries during 2000–2021. It can be found that the number of publications by the United States and China began to increase significantly from 2004 at the earliest. Since 2013, publications from Japan, the UK and Australia in this research field have gradually increased. The cooperation networks between countries in the past two decades have been visualized (Appendix A), which reveals the national cooperation network with the United States and China at the core.

#### 3.1.2. Disciplinary Distribution

**Figure 3:**
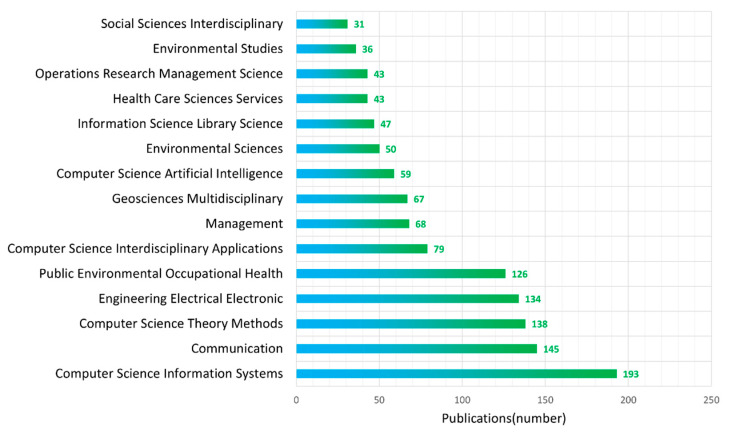
Distributions of popular disciplines. The number of publications from the top 15 disciplines is shown.

This part analyzes the distribution of disciplines of these papers and the 15 disciplines in WoS with the largest number of publications are shown in Figure 3. The figure shows that the discipline of computer science and information system is the discipline with the largest number of articles (193 publications, accounting for 16.8% of the total), followed by communication (145 publications, accounting for 12.7% of the total), computer science theory methods (138 publications, accounting for 12.0% of the total), electrical engineering (134 publications, accounting for 11.7% of the total) and public environmental occupational health (126 publications, accounting for 11.0% of the total). According to the disciplinary distribution in the figure, the majority of articles can be classified into five main disciplines: computer science and information, communication, public health and environment, management and multidisciplinary social sciences. Therefore, we believe that these disciplines make the greatest contribution to the development of disaster information communication research.

#### 3.1.3. Journal Distribution

**Figure 4:**
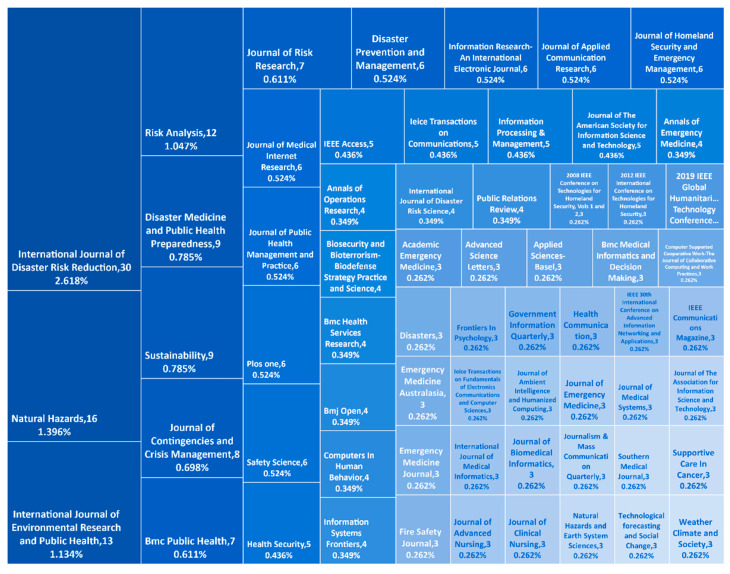
Treemap view of publication distribution. The number of publications of journals and their proportion in the total number of publications are shown.

The journal distribution can indicate the research frontier of the specific field. Journals and proceedings with more than two publications retrieved and 64 journals and proceedings are shown in Figure 4 and the top 10 journals with high publications and citations are listed in Table 1. As these data show, the journals “International Journal of Disaster Risk Reduction”, “Natural Hazards” and “Risk Analysis” have both high publications and citations. These three journals belong to the core journals in the field of disaster and risk research, indicating the dominant position of the discipline in this research field. Regarding the impact factor (IF) from Journal Citation Reports 2021 in WoS, all journals on the list have an index of over 1.00. Further, considering the H-index, all journals have an index of over 40. The H-index is one of the key indicators to evaluate the influence of journals and researchers in academic circles. Combined with the IF and H-index, most studies tend to cite journals with high quality and ranking, indicating that these studies can attract more researchers’ attention and gain greater influence.

#### 3.1.4. Cooperation Network of Institutions

Institute statistics can help to understand the specific situation of research institutions. Table 2 lists the top 20 contributing institutes. Results of contributing institutes show that several effective research institutes in the United States, China, Japan and Canada are of obvious superiority in the number of total publications. Of the top 20 most productive institutions, 7 are from the US, 4 are from Japan, 3 are from China, 3 are from Canada and the other 3 are from Italy, Norway and New Zealand. Researchers from the US and Asia, thus, have the highest levels of interest in the research field of information communication in disasters. From the cooperation network (Appendix B), it can be found that there is a close and steady cooperative relationship between the head institutions. The cooperation network of institutions forms five dense clusters around the head institutions of the United States, China and Japan. American universities, such as UCLA, Pennsylvania State University and the University of Florida, have built bridges for this international cooperation.

### 3.2. Keyword Co-Occurrence Analysis

**Figure 5:**
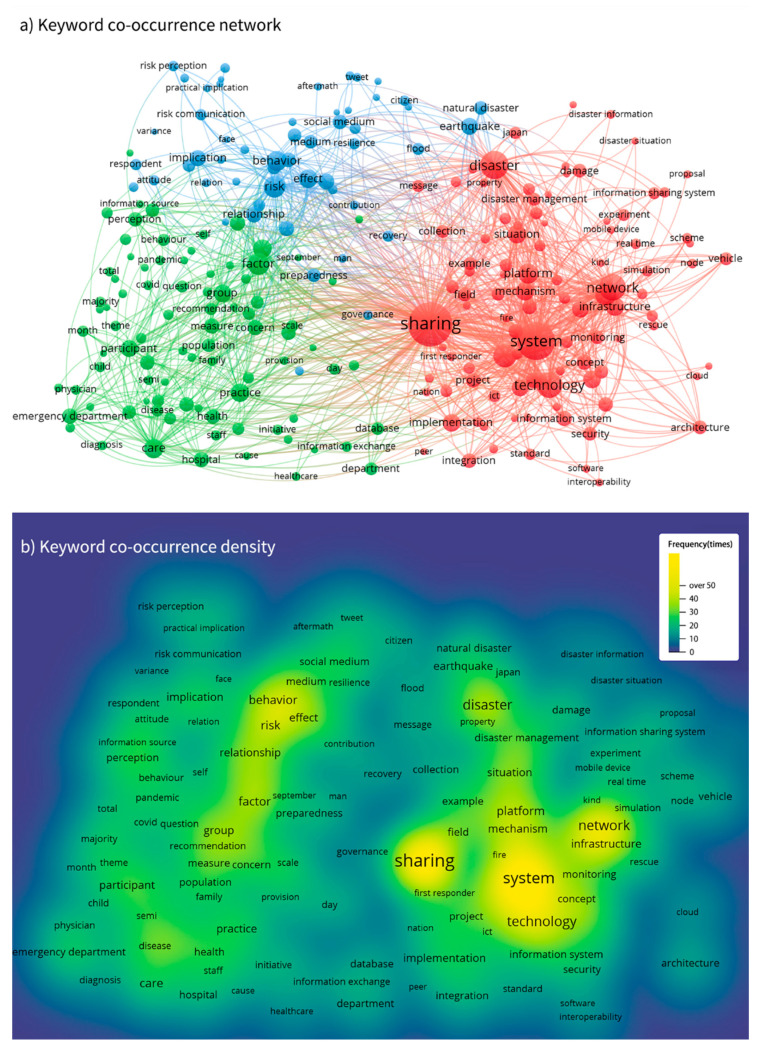
Keyword co-occurrence network and density map.

Keyword co-occurrence analysis is used to explore the hot spots or subject evolution of a certain field or subject. In this study, VOSviewer was used to draw the keyword co-occurrence network and density map (Figure 5) of keywords from 1146 papers in the dataset to intuitively reflect the co-occurrence relationship between high-frequency keywords. It can be concluded from the graph that the topic of sharing behavior, the topic of information system, platform, network and technology, the topic of social media and risk communication and the topic of emergency practice have a high degree of attention.

#### 3.2.1. Keywords Network of Information Communication in Disasters

Figure 5 shows the visualization of the overall co-occurrence network of keywords in this field. From the perspective of keyword frequency, “information sharing” is the keyword with the highest amount of occurrence at 113 times, far exceeding “information seeking” and “communication” with the amount of occurrence at 59 and 83 times. The keyword co-occurrence network from VOSviewer provides three clusters of research orientation in this field: (1) disaster information management and system; (2) risk communication and social media; (3) factors influencing behaviors and perception in disasters.

In terms of disaster information management and system, keywords with high co-occurrence frequency, such as system, technology, network, platform and situation, show that researchers are very concerned about the mechanism and platform system of disaster information communication and management. This part of the research points out the importance of the government and official institutions in the communication of disaster information and discusses the important role of establishing disaster information sharing systems, platforms and mechanisms for emergency management and evacuation. To cope with different kinds of natural disasters, countries in regions prone to natural disasters, such as Japan and China, have established regional information sharing systems (RISSs) to provide disaster information, shelter status and information of required support personnel and support teams, so as to reduce disaster risk and loss [36,37,38]. In this area, Japanese researchers discussed information sharing methods under various information channels and social cooperation modes and made great contributions to the research of information sharing. Some studies also discussed the information-sharing framework during disaster response and recovery for the purpose of reducing the time and cost of emergency response activities [39]. In terms of risk communication and social media, researchers mainly focus on risk communication in disasters, among which the role of social media is focused. The keyword “social media” usually occurs with “management” and “communication”, which shows its value for public crisis communication. Since social media provides a global information exchange environment for disaster preparedness, mitigation, response and recovery, more and more researchers pay attention to the role of social media in natural disasters [40,41,42]. In terms of behaviors and perceptions in disasters, studies focus on exploring the factors that affect people’s risk perception and information exchange behavior in emergencies. Moreover, studies on healthcare information communication, such as studies on people’s health information seeking on the Internet when facing the threat of pandemic [43,44], are the main focuses in this field.

The keyword co-occurrence density map is also offered in Figure 5. The areas with light colors represent the keywords that are more concerned and the areas with deep color represent the keywords that are less concerned. It is easy to find that the cluster of disaster information management and the system is of great interest. Social media and information behavior are another hot research orientation.

#### 3.2.2. Longitudinal Analysis of Keywords

**Figure 6:**
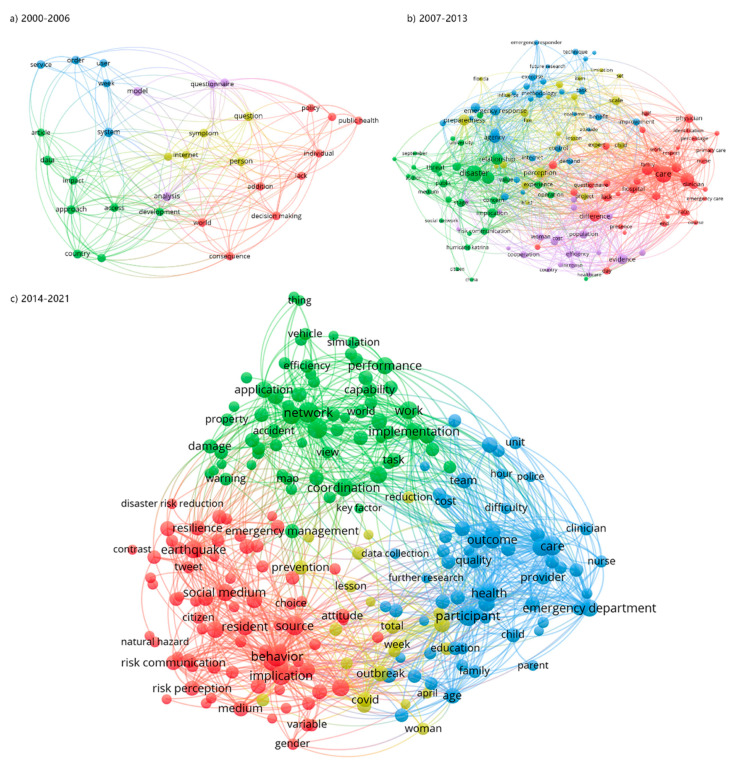
Keyword co-occurrence in 2000–2006, 2007–2013 and 2014–2021.

To investigate the evolution of the keyword network, the entire period of 2000–2021 is divided into three stages, including the initial growth stage (2000–2006), the rapid development stage (2007–2013) and the steady development stage (2014–2021). The results are presented in Figure 6. In the initial growth stage, there are few studies on information communication in disasters and the links between keywords are not closely related. As shown in the diagram, we can find that “development” and “analysis” are at the core of the network, which means studies in this period mainly focus on risk analysis and disaster management capacity building [45,46], as well as disaster response decision-making and policy-making at the national and official institutional levels. “system” and ‘model’ appeared in the first stage, which indicates that studies on disaster information management systems based on computer information technology have received the attention of researchers [47,48]. In the rapid development stage (2007–2013), two research directions closely related to disasters developed rapidly, which are public risk communication in disasters [49,50] and emergency care. The top five keywords in this stage are “social media”, “care”, “system”, “impact” and “risk”. In the second stage, many directions of disaster information dissemination research developed rapidly and many new research topics have emerged. In the steady development stage (2014–2021), the research field of information dissemination in disasters is further expanded, the number of keywords is increasing and the keyword network is more complex. At the same time, three main research clusters were formed at this stage, which are risk communication, emergency care and information network and simulation modeling.

These largely correspond with the three clusters in Figure 6. Thus, it is believed that the main trend of the whole research field is formed at this stage in 2014–2021 [20]. Keywords about information system and relative computer technology appear in the first stage of 2000–2006 and keywords about social media and risk communication appear in the second stage of 2007–2013. However, they have both become the mainstream of research on information communication in disasters. Internet and social media have become the main channels and carriers of disaster information communication and computing has become the main technical way to study information communication mechanisms and risk management [51,52,53,54].

#### 3.2.3. Research Frontier Identification

**Figure 7:**
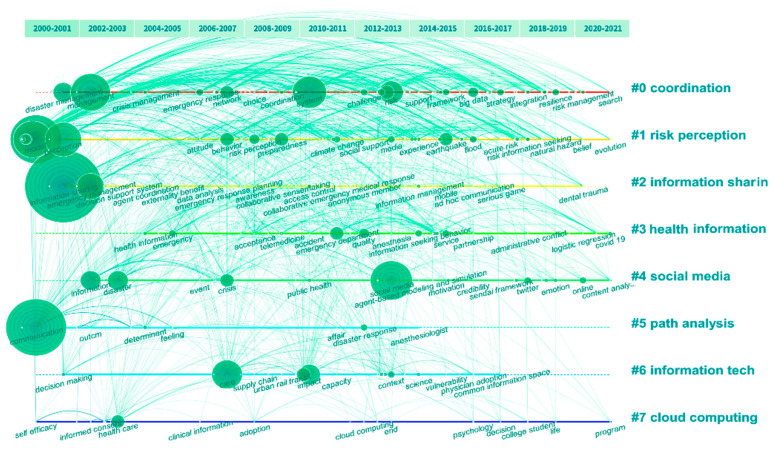
Keyword timeline view of publications on information communication in disasters from 2000 to 2021. The size of the circle represents the importance of the keyword, the links represent the frequency of keyword co-occurrence.

According to the timeline view of keyword co-occurrence (Figure 7), the research trends of information communication in disasters are identified. The timeline view could indicate the evolution of each cluster, which could be useful for research orientation judgement. In the timeline view, the Y-axis represents the number of clusters and the X-axis represents the publication year. The size of the node represents the strength of the node burst and the link between nodes is the co-occurrence between two keywords. All keywords are divided into eight clusters and each cluster contains several keywords.

Cluster #0 (coordination) contains the high-frequency keywords of “network”, “service”, “big data”, “safety”, “crisis management”, etc. The available information infrastructure in emergencies to meet the needs of emergency organizations to obtain information in time is very important for the efficiency of emergency rescue in major events [55,56]. Information sharing among emergency response agencies may be hindered for many reasons, such as information diversion, damage to communication infrastructure and lack of collective situational awareness [57]. If all units cannot cooperate effectively due to information barriers, it will place great pressure on emergency managers and decision makers. In addition, Cluster #2 (information sharing) started near to Cluster #0 and it contains high-frequency keywords of “information sharing”, “emergency management”, “information management”, “awareness”, etc. The correlation between keywords shows that the two clusters are highly correlated. In these clusters, the theoretical focus is on coordination, disaster management roles and information-sharing mechanism.

Cluster #1 (risk perception) contains the high-frequency keywords of “information seeking”, “risk perception”, “preparedness”, “knowledge”, “media”, “experience”, etc. Research in this cluster explored people’s risk perceptions related to their information-seeking behaviors. The role of media and risk communication is also an important part of this cluster. Researchers explored the relationship and reciprocal predictability between risk perception and information seeking regarding natural disasters [58]. In addition, the potential differences in factors, such as knowledge and experience, are discussed [58,59,60]. In this cluster, the theoretical focus is on risk perception attitude, disaster preparedness and information seeking.

Cluster #3 (health information) represents the orientation of emergency information management. It contains the high-frequency keywords of “health”, “emergency department”, “community”, “children”, “intervention”, “follow up”, etc. In this cluster, researchers mainly focus on people’s seeking and sharing behavior of health information [44,61]. Since the worldwide spread of COVID-19, the information seeking and sharing of epidemic and vaccine information has also been widely discussed by researchers [62,63]. Part of the studies discussed the information exchange requirement of healthcare professionals for medical information retrieval, but they have very low relevance to the research topic of this article.

Cluster #4 (social media) indicates the important orientation of crisis communication and social media. It contains the high-frequency keywords of “social media”, “risk”, “crisis”, “emergency”, “Twitter”, “crisis communication”, “online”, “news”, etc. Social media provides a global information exchange environment and platform for disaster preparedness, mitigation, response and recovery [41]; thus, it plays more and more important roles in disaster information communication. Social media could have major functions, including one-way and two-way information sharing, situation awareness, rumor control and emergency decision-making, which help local governments in risk management. Moreover, by discussing people’s information communication behavior on the Internet, the use of public social media and personal social networks is regarded as an important part of crisis management that cannot be ignored [41,51,64]. In addition, Cluster #5 (path analysis) also refers to the research of social networks, but it prefers the studies of communication mechanisms, information technology and systems.

Cluster #5 (path analysis), Cluster #6 (information technology) and Cluster #7 (cloud computing) are all focused on the area of information technology of the Internet and computer science. The high-frequency keywords in Cluster #5 (path analysis) include “technology”, “decision making”, “information technology”, “implementation” and “social network”, which indicates this cluster focuses on the research of social networks and related information technology to help with the decision-making of public crisis management. Cluster #6 (information technology) contains the high-frequency keywords of “communication”, “disaster response”, “determinant”, “outcome” and “consultation”, for which the research direction is biased toward the application of information technology in emergency management. Cluster #7 (cloud computing) contains the high-frequency keywords of “adoption”, “social support”, “cloud computing”, “scale”, “informed consent” and “environment”, representing the direction of information techniques of the network big data information processing [65].

#### 3.2.4. Summary of Keyword Clusters

Clustering analysis of CiteSpace can generate many clusters of keywords and the top-eight clusters are selected to represent the main research content in this field. Comparing the clusters generated, the clusters share great similarities and can be deemed to be overlapping. In the process of analysis, we combine overlapping clusters into the same research direction and finally obtained five main research orientations: (1) public information coordination and information sharing in disaster management; (2) people’s information behavior and perception in disaster; (3) people’s health information communication; (4) risk communication and social media; (5) information technology and information system in disaster management.

The first orientation focuses on information coordination and collaboration in disaster management. Information sharing between emergency rescue units and between officials and victims is the focus of attention in this direction. Disaster management and risk management are the main areas of this research orientation. Moreover, based on keyword cluster analysis, we can further extract sub-themes in this orientation, which include “information sharing system and platform for disaster management” [36,37], “community information coordination network” [66] and “information sharing among disaster responders” [67,68]. These sub-themes further reflect the importance of establishing a high-quality information collaboration between emergency managers, the public and social resources during disasters. Resilience of community information collaboration [69,70], information collaboration between governments and emergency management agencies and various publics [70,71] and supply chain collaboration [72] play an important role in establishing an emergency management framework with long-term disaster response and recovery capabilities in the future.

The second orientation concentrates on public information communication behaviors and risk perception in disasters. In this section, people’s behavior of information seeking, information sharing and communication in disaster were fully discussed. Their risk perception and attitude toward disasters and related activities, determinations that affect people’s behavior and perceptions, such as age, gender, experience and knowledge, are also researched in this orientation. The main sub-themes include “information seeking and sharing in disasters” [2,4], “risk perception on disaster” [58] and “factors causing difference among people in disasters”. In recent years, the relationship between the public’s risk perception and their information search and sharing behavior under long-term disasters [58,63] has been further explored and shows the great value and research potential of establishing a knowledge framework of risk and behavior [73].

The third orientation concentrates on crisis communication and communication in social media. This orientation pays more attention to people’s information seeking, information sharing and communication behavior in disasters on the Internet and social media and studies the communication path and mechanism of crisis information, as well as the corresponding crisis information management mode. With the update of media and the development of social media, researchers are also paying more attention to the relationship between media dependencies and information seeking, as well as preparation behaviors of the public in disasters [74,75]. The main sub-themes include “information communication in social media” [41,76], “Internet information crisis communication” [77] and “communication pathway and mode” [64].

The fourth orientation represents the research of emergency information management. This cluster mainly focuses on people’s seeking and sharing behavior of health information. In addition, the pandemic and vaccine information of COVID-19 is now one of the most concerning research topics in this orientation. Sub-themes in this orientation include “health information communication” [44,61], “pandemic information seeking” [62,63] and “information exchange in medical care” [78].

The last orientation focuses on information technology in disaster and crisis management. The emergence of the Internet and social media has greatly changed people’s information behavior and increased the difficulty of crisis information management. Emergency management using information technology and systems has received increasing attention from scholars and many emergency departments have adopted various advanced information systems to improve their capabilities [20,79]. Information technologies, such as big data, cloud computing, artificial intelligence decision-making and directional push, can be an important part of the emergency response capacity of the government and emergency management agencies [16].

## 4. Discussion

In this paper, statistical analysis of publications in the field of information communication in disasters is performed using the bibliometric method and the knowledge structure is mapped with visualization software. This study further reveals the research status and evolution trends in the field of information communication in disasters. In summary, the conclusions are as follows:

(1) Information communication in disaster research can be divided into the initial growth stage (2000–2006), the rapid development stage (2007–2013) and the steady development stage (2014–2021). The US has the most contributions in the field of information communication in disasters, while China, Japan, the UK, Australia, Italy and Malaysia contribute many publications. However, there is still a lack of global cooperation in this field and the collaboration among researchers needs to be further improved.
(2) As for disciplinary distribution and journal distribution, the journals “International Journal of Disaster Risk Reduction”, “Natural Hazards” and “Risk Analysis” have both high publications and citations. The discipline of computer science information systems, engineering electrical electronics, computer science theory methods and public environmental occupational health have the most contributions in the field of information communication in disasters. Most research belongs to five main disciplines: computer science and information, public environment, management, geosciences and communication.
(3) The cluster analysis of bibliometrics divides the research on disaster information communication into eight main clusters, which are: coordination, risk perception, information sharing, health information, social media, path analysis, information technology and cloud computing. In conclusion, these eight clusters are combined into five research orientations, which are public information coordination in disaster management, public information behavior and perception, health information communication, risk communication and social media and information technology.

These five orientations are regarded to be valuable research areas that researchers in this area should be focusing on. The topic of public information coordination emphasizes the importance of government capacity and mechanism building for emergency response and information management in disasters, which is the basis for disaster risk reduction and disaster recovery. Public information behavior and perception are the core of research in this field, including information communication behavior research at the individual level and group level. Health information communication indicates the information subjects concerned, sought and communicated by people in disasters. The topic of information technology refers to the role of information technology development in disaster information communication research and crisis management. The topic of risk communication and social media emphasizes the importance and attention of social media in people’s information communication behaviors in disasters. This research orientation has attracted much attention in the past decade, surpassing other research topics. Research on the roles of social media in disasters shows its importance in improving government capability for disaster response and management and the potential future value of establishing the online information network, with stronger connectivity and relatively faster information transmission speed [76] to optimize the risk communication.

### Limitations

In this study, a bibliometric analysis was conducted in the field of information seeking, information sharing and communication in disasters and a comprehensive overview of this field is mapped and the knowledge structure and future trends are visualized. However, some limitations and suggested improvements should be acknowledged.

Firstly, the WOS database is used to extract the original data from the literature. The lack of year, source, keywords and abstract in some of the literature may lead to a deviation in research results, which needs more careful manual processing to improve the accuracy. To solve this problem, a more comprehensive database combination should be considered in future research. Secondly, the VOSviewer and CiteSpace software used in this study have limitations in the algorithm of keyword network analysis, due to the limitations in flexibility and expansibility of the algorithm. Therefore, expanding the publication data analysis function by introducing advanced R tools according to the needs of different research topics will help to obtain broader and more accurate data results. Next, the accuracy of bibliometric analysis depends on the academic publications and topic words the researchers use, which may lead to differences in bibliometric data of different studies under the same topic. Lastly, the selection dimensions of the core keywords of disaster information communication research publications may not be comprehensive enough to fully reflect the valuable research hotspots under the topic. In the future, the weight of core keywords, frontier institutions and core journals in the field can be further allocated to obtain more convincing and valuable research results. Future works require in-depth research on the important research orientations shown in the research field of information communication in disasters and further analyze its research status and trends for a specific research topic. As for the bibliometric study, it is necessary to introduce qualitative research of experts’ views, rather than relying on software techniques. Moreover, the optimization of the visualization of results will contribute to the comprehensibility and dissemination of research.

## 5. Conclusions

In this study, an analysis of the current research landscape in the field of information communication behavior in disasters is conducted by identifying different trends and patterns in publications and citations, showing the global collaboration network with the U.S. and China as the core. Discipline analysis shows the vigorous development trends of disaster management, information science, communication, engineering and their collaboration. Keyword analysis within the research field is then conducted to investigate popular keywords and their evolution over time. The trend of publications in the past two decades reveals three main stages in the whole development process and the research on risk communication, emergency care and information network and simulation modeling that is concerned at the current stage is emphasized. A keyword-based cluster analysis is conducted to examine the major research orientations. The five research orientations of public information coordination in disaster management, public information behavior and perception, health information communication, risk communication and social media and information technology in disasters show their important value and development prospects in the research of information communication behavior in disasters. The findings from the analysis, thus, reveal the mapping of the research on information communication behavior in disasters and offer an extensive overview for scholars, enabling them to conduct further studies.

## Figures and Tables

**Table 1:** Journal distribution (TOP 10).

	Journals	Publications	Citations	IF 2021	H Index
1	International Journal of Disaster Risk Reduction	30	291	4.842	58
2	Natural Hazards	16	243	3.158	114
3	International Journal of Environmental Research and Public Health	13	123	4.614	138
4	Risk Analysis	12	385	4.302	139
5	Disaster Medicine and Public Health Preparedness	9	46	5.556	47
6	Sustainability	9	28	3.889	109
7	Journal of Contingencies and Crisis Management	8	37	3.420	55
8	BMC Public Health	7	116	4.135	159
9	Journal of Risk Research	7	52	5.346	65
10	Disaster Prevention and Management	6	78	1.813	57

Note: The journal distribution of the 1146 publications is analyzed and the TOP 10 contributing journals are displayed according to the number of publications in the journal. The impact factors in 2021 are from the Journal Citation Reports 2021 in WoS. The H-index is from the Scimago Journal Rankings database and the H-index of these journals are shown.

**Table 2:** Top 20 contributing institutions.

Institutions	Country	Origin Year	Publications
Iwate Prefectural Univ	Japan	2010	16
Arizona State Univ	US	2005	11
Keio Univ	Japan	2010	10
Univ Colorado	US	2007	10
Penn State Univ	US	2000	9
Johns Hopkins Univ	US	2002	9
Univ Agder	Norway	2015	8
Tsinghua Univ	China	2012	7
Beijing Jiaotong Univ	China	2008	6
Fukuoka Inst Technol	Japan	2013	6
Univ Ottawa	Canada	2009	6
Tohoku Univ	Japan	2006	5
Univ Insubria	Italy	2013	5
Univ Auckland	New Zealand	2016	5
Univ Washington	US	2005	5
UCL	US	2005	5
Univ British Columbia	Canada	2004	5
Univ Sci & Technol China	China	2016	5
Univ Florida	US	2012	5
Univ Waterloo	Canada	2001	4

Note: The institution distribution of the 1146 publications is analyzed and the TOP 20 contributing journals are displayed according to the number of publications in the journal. The origin year represents the earliest publication year of the institute in the 1146 publications.

**Figure A1:**
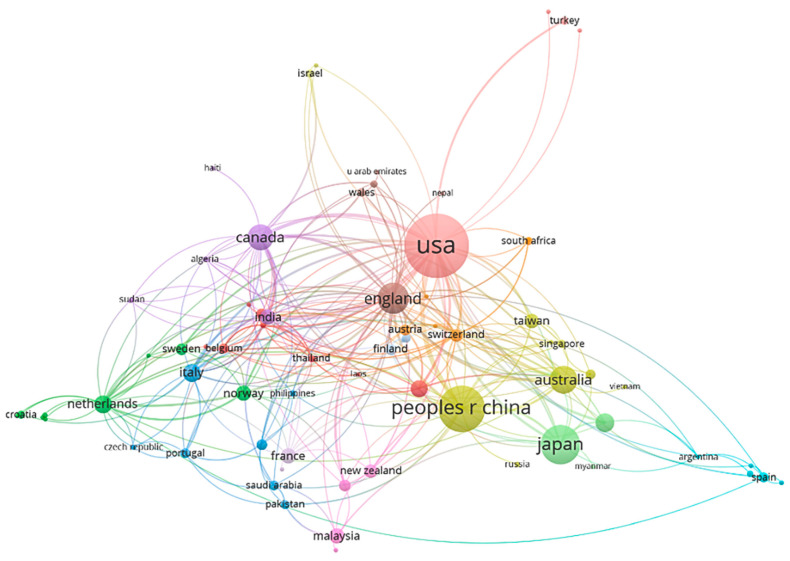
Collaboration network map of main countries.

**Figure A2:**
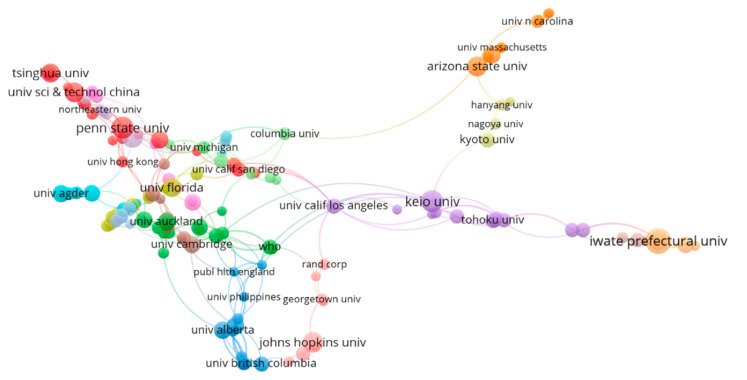
Collaboration network map of main institutions.

## Data Availability

The data presented in this study are available on request from the corresponding author.
